# DNA Damage–Induced Bcl-x_L_ Deamidation Is Mediated by NHE-1 Antiport Regulated Intracellular pH

**DOI:** 10.1371/journal.pbio.0050001

**Published:** 2006-12-19

**Authors:** Rui Zhao, David Oxley, Trevor S Smith, George A Follows, Anthony R Green, Denis R Alexander

**Affiliations:** 1 Laboratory of Lymphocyte Signalling and Development, The Babraham Institute, Babraham, Cambridge, United Kingdom; 2 Protein Technologies Laboratory, The Babraham Institute, Babraham, Cambridge, United Kingdom; 3 Department of Haematology, University of Cambridge, Hills Road, Cambridge, United Kingdom; St. Jude Children's Research Hospital, United States of America

## Abstract

The pro-survival protein Bcl-x_L_ is critical for the resistance of tumour cells to DNA damage. We have previously demonstrated, using a mouse cancer model, that oncogenic tyrosine kinase inhibition of DNA damage–induced Bcl-x_L_ deamidation tightly correlates with T cell transformation in vivo, although the pathway to Bcl-x_L_ deamidation remains unknown and its functional consequences unclear. We show here that rBcl-x_L_ deamidation generates an iso-Asp^52^/iso-Asp^66^ species that is unable to sequester pro-apoptotic BH3-only proteins such as Bim and Puma. DNA damage in thymocytes results in increased expression of the NHE-1 Na/H antiport, an event both necessary and sufficient for subsequent intracellular alkalinisation, Bcl-x_L_ deamidation, and apoptosis. In murine thymocytes and tumour cells expressing an oncogenic tyrosine kinase, this DNA damage–induced cascade is blocked. Enforced intracellular alkalinisation mimics the effects of DNA damage in murine tumour cells and human B-lineage chronic lymphocytic leukaemia cells, thereby causing Bcl-x_L_ deamidation and increased apoptosis. Our results define a signalling pathway leading from DNA damage to up-regulation of the NHE-1 antiport, to intracellular alkalanisation to Bcl-x_L_ deamidation, to apoptosis, representing the first example, to our knowledge, of how deamidation of internal asparagine residues can be regulated in a protein in vivo. Our findings also suggest novel approaches to cancer therapy.

## Author Summary

Cell survival and cell death (apoptosis) are controlled by a finely tuned ensemble of pro-survival and pro-apoptotic proteins. When the two types of protein are balanced, cells survive. But if the pro-survival proteins dominate, there is a danger that cells with damaged DNA will stay alive, leading to malignancy. One of the key pro-survival proteins, Bcl-x_L_, acts by blocking the actions of pro-apoptotic proteins. We show here that DNA damage results in an important modification of Bcl-x_L_. Specifically, when the amide groups are removed from two critical asparagine (amino acid) residues, Bcl-x_L_ can no longer block pro-apoptotic proteins, leading to cell death. Surprisingly, Bcl-x_L_ deamidation is catalysed not by an enzyme, but by increased pH inside the cell due to the up-regulation of an NHE-1 transporter that moves positive ions across the cell membrane. Indeed, artificially increasing pH causes Bcl-x_L_ deamidation and apoptosis in the absence of initial DNA damage. Exploring this novel pathway may ultimately suggest approaches to cancer therapy, especially when malignant cells are resistant to chemotherapy or radiotherapy.

## Abstract 3

Until now, the mechanisms and functional implications for DNA damage-induced Bcl-x_L_ deamidation were unknown. Here the authors provide important new insights into this phenomenon and its impact on cell survival.

## Introduction

The deamidation of internal asparaginyl and glutaminyl protein residues has attracted increasing attention over the past decade as a modification leading to significant changes in protein function [1,2]. The protein deamidation rates of more than 18,000 proteins have been computed, containing 230,000 individual asaparaginyl residues, generating Asn half-lives of less than 1 d to 50 y or more [3,4]. Protein deamidation has broad biological implications, ranging from changes in the specificity of antigen presentation [5], to modifications in eye lens proteins [6], to the activation of RhoA by cytotoxic necrotizing factor [7], to aging [1], to name but a few examples.

The deamidation of Gln proceeds both enzymatically and nonenzymatically in physiological systems, whereas only the nonenzymatic deamidation of internal Asn residues has been reported, involving conversion to Iso-Asp:Asp in a ratio of about 3:1, with the precise ratio depending on the environment of the Asn residue [1,8]. Deamidation of both Gln and Asn residues in vitro can be greatly accelerated by exposure to either acid or alkaline pH, with minima in the range pH 4–6. Until recently, it was assumed that Asn protein deamidation rates in vivo were set up by a “fixed clock” that was defined only by the primary, secondary, and tertiary structures of proteins that specified the half-life of the particular Asn residue in question. However, this view has been radically changed by the recent observation that DNA damage induces the relatively rapid deamidation of the pro-survival protein Bcl-x_L_ in an osteosarcoma cell line system [9], indicating that the deamidation “clock”, far from being fixed, is a dynamic process that can be regulated in vivo by biologically critical events. Bcl-x_L_ deamidation in response to DNA damage occurs at two internal Asn residues (Asn^52^ and Asn^66^), causing a characteristic retardation on SDS-polyacrylamide gel eletrophoresis (PAGE) gels [9–12]. Initial work from the Weintraub laboratory suggested that when Asn^52^ and Asn^66^ are both mutated to Asp, then Bcl-x_L_ loses its ability to bind to the BH3-only pro-apoptotic protein Bim, thereby providing a putative linkage between DNA damage and apoptosis [9]. However, a secondary mutation was later identified, which, when corrected, enabled the N52D/N66D Bcl-x_L_ to bind Bim, casting doubt on this interpretation [13].

Using a different model system, we have previously implicated the oncogene-mediated inhibition of DNA damage-induced Bcl-x_L_ deamidation in the transformation of murine thymocytes [14,15]. Our transgenic mouse model of T cell lymphoma was generated by crossing mice lacking expression of the CD45 tyrosine phosphatase with a line expressing a nononcogenic level of the mutant lck^F505^ tyrosine kinase [16]. All the *CD45^−/−^lck^F505^* progeny develop aggressive T cell lymphomas at the early CD4^−^CD8^−^ stage of thymic development, typically at 5–12 wk of age. The absence of CD45-mediated dephosphorylation results in hyperphosphorylation of positive regulatory p56^lck^ pTyr-394, causing hyperactivation of the kinase and triggering oncogenesis [15]. The model enables the investigation of the earliest oncogenic events in primary pretumourigenic thymocytes. Inhibition of DNA repair in *CD45^−/−^lck^F505^* mice leads to DNA damage, genomic instability, and chromosomal aberrations detectable in primary CD4^−^CD8^−^ thymocytes before transformation. Despite a normal p53 response, DNA damage–induced apoptosis is suppressed in pretumourigenic thymocytes, correlating with the inhibition of Bcl-x_L_ deamidation, the preservation of Bcl-x_L_ binding to Bim, and the inhibition of cytochrome c release and the apoptotic caspase execution cascade. Therefore, we proposed that Bcl-x_L_ deamidation is a critical switch in oncogenic kinase-induced T cell transformation, and we suggested that Bcl-x_L_ deamidation to an Iso-Asp^52^/Iso-Asp^66^ version, rather than the mutant N52D/N66D version investigated by the Weintraub laboratory, might be the key step in disabling the antiapoptotic functions of the protein [14,15].

Neither in the osteosarcoma cell line work [9] nor in our own work based on primary thymocytes [15] has there been any indication as to how DNA damage might induce Bcl-x_L_ deamidation. Neither have there been previous reports in the literature showing how protein Asn deamidation in general might be regulated in vivo; we address here this question. We confirm that Bcl-x_L_ deamidation does indeed destroy its ability to sequester pro-apoptotic proteins such as Bim and Puma, thereby establishing a clear molecular link between DNA damage, Bcl-x_L_ deamidation, and apoptosis. Surprisingly, DNA damage–triggered deamidation in primary wild-type cells is mediated not enzymatically, but by intracellular alkalinisation caused by increased expression of the NHE-1 Na^+^/H^+^ exchanger (antiport), events blocked by expression of the oncogenic tyrosine kinase (OTK). In the case of either murine or human cancer cells, enforced alkalinisation triggers Bcl-x_L_ deamidation, crippling its ability to provide protection from the pro-apoptotic consequences of DNA damage, thereby indicating possible novel approaches to cancer therapy.

## Results

### DNA Damage–Induced Bcl-x_L_ Deamidation Does Not Depend on Mitochondrial Apoptosis

**Figure 1:**
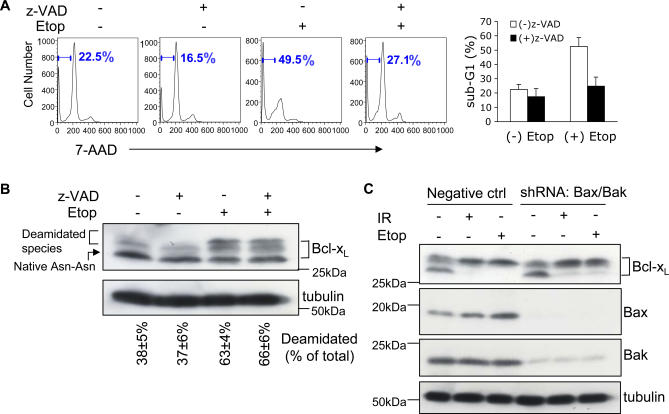
DNA Damage–Induced Bcl-x_L_ Deamidation Is Mitochondrial Apoptosis–Independent (A) Wild-type thymocytes were pre-incubated with or without Z-VAD-fmk (200 μM), and were then cultured with or without etoposide for 24 h, harvested, and apoptosis was measured by measuring the sub-G1 peak by flow cytometry. The histograms (right panel) represent mean values ± SD (*n* = 3). (B) Aliquots of the cells from (A) incubated in the presence or absence of Z-VAD-fmk (200 μM) were analysed for the expression of Bcl-x_L_ and tubulin (as loading control) by immunoblotting. The upper and lower bands of Bcl-x_L_ were quantified and expressed as a percentage of total Bcl-x_L_. The percentages shown below each lane are means ± SD (*n* = 3). (C) Plasmids of shRNA Bax (GFP) and shRNA Bak (DsRed) were cotransfected into purified DN thymocytes using an Amaxa nucleofactor kit. 48 h later, GFP^+^ DsRed^+^ cells were purified by flow cytometry and treated with etoposide (Etop, 25 μM) for 30 h or exposed to irradiation (IR, 5 Gy) followed by 30 h in culture. DN thymocytes transfected with negative control plasmids were treated in parallel. Cells were then processed for immunoblotting with Bcl-x_L_ antibody. The immunoblot was reprobed for Bax and Bak to check the efficiency of gene knockdown. Tubulin was also reprobed as a loading control.

An important consideration is whether DNA damage–induced Bcl-x_L_ deamidation in murine thymocytes is a cause or consequence of thymic apoptosis. Figure 1 shows that whereas the addition of the caspase inhibitor Z-VAD-fmk, as expected, inhibited DNA damage–induced apoptosis in murine thymocytes (Figure 1A), no inhibition of DNA damage–induced Bcl-x_L_ deamidation was observed in cell aliquots taken from the same thymic cultures (Figure 1B). It is known that in the absence of Bax and Bak, BH3-only proteins are unable to induce apoptosis [17]. We therefore used short hairpin RNA (shRNA) to deplete Bax and Bak from CD4^−^CD8^−^ (double-negative, DN) thymocytes, confirmed that depletion was sufficient to block caspase 9 cleavage (Figure S1A), and showed that DNA damage–induced Bcl-x_L_ deamidation proceeded normally in the absence of Bax and Bak (Figure 1C). We also showed that Bcl-x_L_ deamidation was clearly detectable within 3–6 h after the instigation of DNA damage, and proceeded in parallel with increased apoptosis (Figure S1B and S1C). These results show that Bcl-x_L_ deamidation is not caused by mitochondrial apoptosis and are consistent with a role for deamidation upstream of the apoptotic executor pathway. Further data presented below establish a more direct causal relationship between Bcl-x_L_ deamidation and apoptosis in DNA damaged thymocytes.

### Bcl-x_L_ Deamidation In Situ Involves Conversion of Asn^52^/Asn^66^ to Iso-Asp^52^/Iso-Asp^66^, Preventing Sequestration of Bim and Puma

We previously noted that whereas the ability of Bcl-x_L_ to bind Bim was ablated in control thymocytes exposed to DNA damage, it was strikingly retained in pretumourigenic *CD45^−/−^lck^F505^* thymocytes, tightly correlating with the resistance to Bcl-x_L_ deamidation noted in these cells [15]. However, work from the Weintraub laboratory suggests that deamidated Bcl-x_L_ still binds Bim [13], thereby casting doubt on the model that Bcl-x_L_ deamidation triggers apoptosis. Because the sequestration of BH3-only proteins by Bcl-x_L_ is thought to explain its anti-apoptotic function [18], resolution of this question is clearly important for establishing a molecular link between DNA damage and apoptosis. We therefore carried out a series of cellular and biochemical experiments to address this key point.

**Figure 2:**
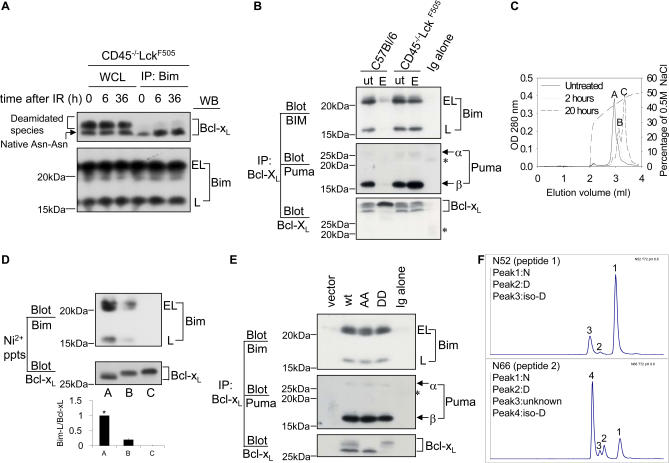
Deamidation Disrupts the Sequestration of BH3-Only Proteins by Bcl-x_L_ (A) Bim binds to the native (Asn-Asn) but not deamidated forms of Bcl-x_L_. Wild-type (C57BL/6) thymocytes (1.5 × 10^7^) were exposed to 5 Gy irradiation (IR) and then maintained in culture for the times shown, after which cells were lysed and either separated as whole cell lysates (WCL) or as Bim immunoprecipitates, followed by immunoblotting for either Bcl-x_L_ or for Bim. Bim migrates as “extra-long” (EL) or “long” (L) forms. (B) Bcl-x_L_ was immunoprecipitated from lysates derived from purified DN thymocytes treated with/without etoposide (ut/E), followed by immunoblotting for Bim or Puma. The asterisk indicates the light chain of the Bcl-x_L_ antibody used for immunoprecipitation. (C) Anion exchange chromatography of purified rBcl-x_L_. Sample A was untreated; samples B and C were exposed to pH 8.8 at 37 °C for 2 h and 20 h, respectively. The Figure illustrates superimposed elution profiles for each sample. Peaks A, B, and C had molecular masses of 25, 015.6; 25, 016.4, and 25, 017.2, respectively. (D) Bim binds to native but not to deamidated rBcl-x_L_. The three different forms of Bcl-x_L_ (A, B, and C) purified by anion-exchange column chromatography shown in (C) were incubated in wild-type thymic lysates (1.5 × 10^7^ cell equivalents) at 4 °C for 2 h and then precipitated using nickel beads. The precipitated products were immunoblotted for Bim and Bcl-x_L_. Quantification of the Bim-L/Bcl-x_L_ ratios ± SD from three independent experiments is shown in the histogram, with the lane A ratio normalised to 1 (*). (E) Primary thymocytes were retrovirally transduced with empty vector or Bcl-x_L_ constructs (wild-type, N52A-N66A, or N52D-N66D). Bcl-x_L_ was immunoprecipitated from lysates derived from 1.5 × 10^6^ sorted GFP-positive cells per lane, followed by immunoblotting for Bim or Puma. Note that in the vector lane, at this exposure endogenous Bcl-x_L_ is not visible because of the small number of cells used. The asterisk indicates the light chain of the Bcl-x_L_ antibody used for immunoprecipitation. (F) Peptides SDVEENRTEAPEGTESEMETPSAINGNPSW (peptide 1) and HLADSPAVNGATGHSSSL (peptide 2), and the corresponding deamidated forms, containing the putative deamidation sites N52 and N66, respectively, were generated by digestion of rBcl-x_L_ with chymotrypsin. The chromatographic conditions used for the separation of the peptides in the LC-MS analyses were optimised so as to resolve the Asn, Asp, and iso-Asp forms of peptides 1 and 2. The Asp and iso-Asp forms of the two peptides were identified by spiking an aliquot of a digestion mixture with Asp- or iso-Asp–containing synthetic peptides prior to LC-MS (Figure S3). The chromatograms show LC-MS analyses at time point 72 h of the rBcl-xl base treatment. For both peptides, the major deamidation product is the iso-Asp form; the iso-Asp:Asp ratios are approximately 10:1 for N52 and 5:1 for N66. The unknown peak 3 in peptide 2 could be an isomer of peak 2 or peak 4.

Figure 2A shows that Bcl-x_L_ measured in whole cell lysates from pretumourigenic *CD45^−/−^lck^F505^* murine thymocytes is resistant to deamidation following γ irradiation, consistent with our previous findings [15]. Immunoprecipitation of the pro-apoptotic protein Bim, followed by immunoblotting for Bcl-x_L_, revealed that Bim sequestered only the N52/N66 Bcl-x_L_ and failed to bind the slower migrating deamidated protein (Figure 2A, upper panel), although the amount of Bim in each immunoprecipitate was comparable (Figure 2A, lower panel). Because the BH3-only protein Puma, not Bim, plays a major role in DNA-damage triggered apoptosis [19,20], we also showed that both Puma and Bim are found in Bcl-x_L_ immunoprecipitates from etoposide treated *CD45^−/−^Lck^F505^* thymocytes, whereas sequestration is ablated in wild-type cells, correlating with Bcl-x_L_ deamidation (Figure 2B). A comparable result was obtained when Puma immunoprecipitates were blotted for Bcl-x_L_ (Figure S2A). Therefore, deamidated Bcl-x_L_ appears unable to sequester BH3-only proteins.

To confirm the results using intact thymocytes, we carried out in vitro biochemical experiments. Recombinant purified His-tagged Bcl-x_L_ was exposed to alkaline conditions to cause partial deamidation and separated by anion-exchange chromatography into three peaks (Figure 2C, peaks A, B and C). Mass spectrometric analysis revealed an increase of 1 Da for peak B relative to peak A, and a further increase of 1 Da for peak C relative to peak B (Figure 2C). On SDS-PAGE gels, peak A Bcl-x_L_ migrated slightly faster than the more acidic peaks B and C (Figure 2D), reproducing the characteristic profile of N52/N66 Bcl-x_L_ and its deamidated versions found in our cellular studies (Figure 1B). It has already been demonstrated that these migratory shifts are not caused by phosphorylation [9,12]. In fact, deamidation of a single Asn increases protein mass by 1 Da, at the same time increasing its net negative charge, confirming that the shifts are due to deamidation. Importantly, when the three species of rBcl-x_L_ were tested for their ability to bind to Bim in wild-type thymic lysates, only peak A bound Bim effectively, whereas binding to peak B rBcl-x_L_ was reduced by 88% ± 2% and completely ablated using peak C rBcl-x_L_ (Figure 2D, upper panel). Figure 2E shows that the Asp^52^/Asp^66^ version of Bcl-x_L_, or the Ala^52^/Ala^66^ version that cannot be deamidated, does still bind both Bim and Puma, consistent with the correction published by the Weintraub laboratory [13]. We therefore determined whether rBcl-x_L_ Asn^52^ and Asn^66^ convert mainly to Asp or to iso-Asp upon alkali treatment. Consistent with previous results [8], Figure 2F and Figure S3 show that the ratios of iso-Asp:Asp conversion for Asn^52^ and Asn^66^ are 10:1 and 5:1, respectively. Kinetic analysis revealed that deamidation of Asn^66^ to iso-Asp is much faster than for Asn^52^ (unpublished data).

Taken together, our results show that conversion of Bcl-x_L_ Asn^52^ and Asn^66^ to iso-Asp, but not Asp, prevents sequestration of BH3-only proteins. Peak B represents rBcl-x_L_ deamidated at either Asn^52^ or Asn^66^, whereas peak C is deamidated at both sites (Figure 2C and 2D). Deamidation to iso-Asp causes greater perturbations of protein structure than conversion to Asp [1,8], presumably explaining the loss of BH3-only protein binding.

### DNA Damage–Induced Bcl-x_L_ Deamidation and Apoptosis Is Mediated by Intracellular Alkalinisation

**Figure 3:**
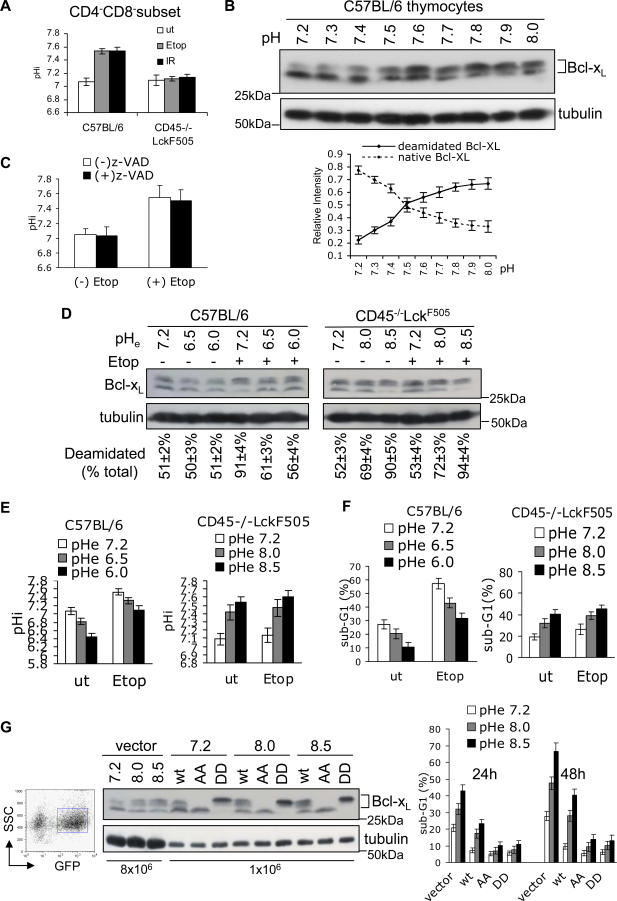
DNA Damage Causes Intracellular Alkalinisation and Subsequent Bcl-x_L_ Deamidation (A) Intracellular alkalinisation occurs following DNA damage in wild-type but not in pretumourigenic *CD45^−/−^Lck^F505^* thymocytes. Cells were treated with etoposide (Etop) for 20 h or exposed to 5 Gy of irradiation (IR) and then maintained in culture for 20 h. pH_i_ was measured using SNARF by FACS in the gated live CD4^−^CD8^−^ subset. The histograms represent mean values ± SD (*n* = 5). (B) Enforced intracellular thymic alkalinisation causes Bcl-x_L_ deamidation. Wild-type thymocytes were maintained in RPMI-1640/10% bovine fetal calf serum buffered at the indicated pH with Tris-HCl for 20 h in the presence of 20 μM monensin prior to lysis and immunoblotting for Bcl-x_L_. To minimize any deamidation produced during the gel-running process, the resolving gel buffer was adjusted to pH 8.0 in this experiment. The mean ratio of the lower band (native Bcl-x_L_) or upper band (deamidated Bcl-x_L_) to the total (upper plus lower bands) is shown in the graph (lower panel). The error bars represent SD (*n* = 3). Note that deamidation becomes prominent at pH 7.5. (C) Aliquots of the cells from Figure 1A incubated in the presence or absence of Z-VAD-fmk (200 μM) were analysed for pH_i_, The histograms represent mean values ±SD (*n* = 3). (D) Wild-type or *CD45^−/−^Lck^F505^* pretumourigenic thymocytes were cultured for 24 h in media at the pH shown without monensin, with or without etoposide, and then analysed for Bcl-x_L_ deamidation by immunoblotting. The upper and lower bands were quantified and the percentage of upper bands in total Bcl-x_L_ calculated. The percentages shown below each lane are means ± SD (*n* = 5). (E) Aliquots of cells used in (D) were assessed for pH_i_ by FACS. The histograms show the pH_i_ of live gated CD4^−^CD8^−^ thymocytes from five independent experiments ±SD. The pH_e_ values refer to the pH values of the extracellular media. (F) Apoptosis of aliquots of the cells from (D) was analysed by FACS. The histogram shows the sub-G1 peak (%) of CD4^−^CD8^−^ thymocytes from five independent experiments ± SD. (G) Wild-type (wt), N52A-N66A (AA), N52D-N66D (DD) Bcl-x_L_, and empty vector were retrovirally transduced into thymocytes. GFP-positive cells were FACS sorted (left panel) and cultured in media with the pH_e_ shown for 24 h or 48 h, then processed for immunoblotting with Bcl-x_L_ antibody (middle panel). Note that 8 × 10^6^ and 1 × 10^6^ cell equivalents were loaded per lane for the empty vector (lanes 1–3) and Bcl-x_L_ (lanes 4–12) transfectants, respectively, such that the endogenous Bcl-x_L_ is invisible in lanes 4–12. The histogram (right panel) shows mean apoptosis (sub-G1) values ±SD generated from five independent experiments.

Until now, the in vivo mechanism for the deamidation of internal protein Asn residues has not been described for any protein. Because protein Asn deamidation is accelerated by increased pH in vitro, we investigated intracellular pH change (pH_i_) as a possible regulatory mechanism in thymocytes. Figure 3A shows that after DNA damage, the pH_i_ of live wild-type CD4^−^CD8^−^ thymocytes increased to 7.55, whereas no change was observed in pretumourigenic cells. But is that increase sufficient to cause Bcl-x_L_ deamidation? To address this question, we incubated wild-type thymocytes in the pH range of 7.2–8.0 for 20 h in the presence of the Na^+^ ionophore monensin to ensure complete equilibration of pH_i_ and extracellular pH (pH_e_), and to neutralize acidic intracellular compartments [21], and we then assessed the extent of Bcl-x_L_ deamidation. Figure 3B shows that whereas only 22.5% ± 3.2% was deamidated at pH 7.2, this increased to 56.1% ± 3.8% at pH 7.6 and 67.0% ± 4.5% at pH 8.0. Therefore, a rise in pH_i_ comparable with that observed after DNA damage (Figure 3A) is sufficient to cause substantial deamidation. Furthermore, the addition of Z-VAD-fmk to thymic cultures following DNA damage did not inhibit their alkalinisation (Fig. 3C), showing that the rise in pH_i_ is not downstream of caspase activation. To investigate Bcl-x_L_ deamidation, pH_i_, and apoptosis in parallel, we manipulated pH_i_ values artificially by incubating cells at varying pH_e_ values in the absence of monensin. The left panel of Figure 3D shows that when DNA damage was induced in wild-type thymocytes, Bcl-x_L_ deamidation could be largely prevented by artificially maintaining the pH_i_ at 7.1 (value shown in Figure 3E, left panel), thereby reducing the percentage of apoptotic CD4^−^CD8^−^ thymocytes by 2-fold relative to those incubated at physiological pH (Figure 3F, left panel). Conversely, Figure 3D (right panel) shows that the resistance to Bcl-x_L_ deamidation observed in DNA-damaged pretumourigenic thymocytes could be completely overcome by artificially increasing the pH_i_ to 7.55 or above (Figure 3E, right panel), correlating with a 2-fold increase in the percentage of apoptotic CD4^−^CD8^−^ thymocytes relative to those incubated at physiological pH (Figure 3F, right panel). Interestingly, enforced alkalinisation alone in the absence of DNA damage caused a marked increase in Bcl-x_L_ deamidation in the OTK expressing thymocytes (Figure 3D, right panel), with a concomitant increase in apoptosis (Figure 3F, right panel), albeit at a level lower than with DNA damage, perhaps reflecting the somewhat lower pH_i_ values achieved under these conditions (Figure 3E, right panel).

We considered that the tight correlation between pH_i_, Bcl-x_L_ deamidation, and apoptosis might nevertheless be coincidental and that enforced alkalinisation might be inducing apoptosis by a mechanism independent of Bcl-x_L_ deamidation. Mutant Bcl-x_L_ Ala^52^/Ala^66^ or Asp^52^/Asp^66^, both of which sequester BH3-only proteins (Figure 2E), were therefore over-expressed in wild-type CD4^−^CD8^−^ thymocytes by retroviral transduction prior to enforced alkalinisation by incubation in media at pH 8.0 or 8.5. Figure 3G (middle panel) shows that, as expected, the Ala^52^/Ala^66^ mutant migrates as the lower nondeamidated version of Bcl-x_L_, whereas Asp^52^/Asp^66^ migrates as the more negatively charged deamidated version. Interestingly, in the cells expressing these mutant forms of Bcl-x_L_, the apoptosis induced by enforced alkalinisation was reduced 4-fold compared to cells transduced with empty vector, or more than 2-fold in comparison with the wild-type protein (Figure 3G, right panel), which of course undergoes deamidation in response to alkali treatment. These results show that Bcl-x_L_ in a version able to sequester BH3-only proteins protects thymocytes from an enforced increase in pH_i_. Nevertheless, protection was not absolute, suggesting that Bcl-x_L_ may not be the only mechanism protecting cells from apoptosis triggered by alkalinisation. As a further control, we have confirmed that Bcl-x_L_ isolated from wild-type thymocytes exposed to a high pH buffer can no longer sequester Bim (Figure S2B), thereby mimicking the effects of DNA damage (Figure 2A).

Taken overall, these results demonstrate that intracellular alkalinisation following DNA damage is both necessary and sufficient for nonenzymatic Bcl-x_L_ deamidation, that the oncogenic suppression of Bcl-x_L_ deamidation in pretumourigenic thymocytes is caused by inhibition of alkalinisation, and that versions of Bcl-x_L_ competent for BH3-only protein sequestration are sufficient per se to protect cells from apoptosis at alkaline pH_i_.

### DNA Damage–Induced Alkalinisation, Bcl-x_L_ Deamidation, and Apoptosis are Mediated by Increased NHE-1 Antiport Expression

**Figure 4:**
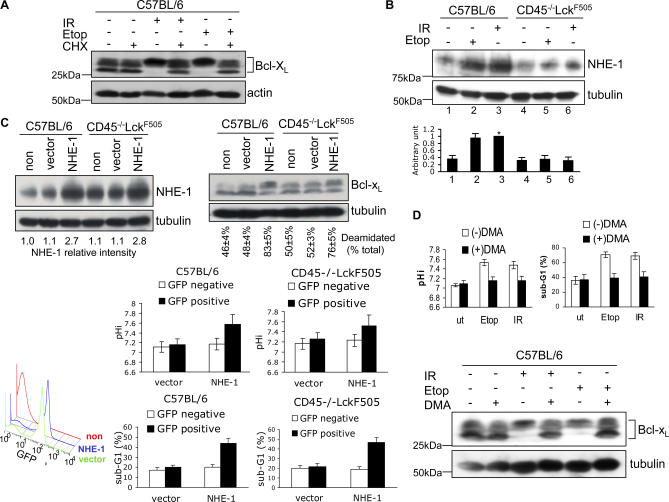
Bcl-x_L_ Deamidation Induced by DNA Damage Involves Up-Regulation of the NHE-1 Na/H Antiport (A) Bcl-x_L_ deamidation induced by DNA damage requires de novo protein synthesis. Wild-type thymocytes were either treated with etoposide for 24 h (Etop), or exposed to 5 Gy of irradiation (IR) and then maintained in culture for 24 h, with or without 0.5 μM cycloheximide (CHX). Cell lysates were processed by immunoblotting for Bcl-x_L_ or β-actin (loading control). (B) DNA damage causes up-regulation of NHE-1 in wild-type but not in *CD45^−/−^Lck^F505^* thymocytes. Wild-type or *CD45^−/−^Lck^F505^* thymocytes were either treated with etoposide (Etop) for 5 h, or exposed to 5 Gy of irradiation (IR) and then maintained in culture for 5 h before immunoblotting for NHE-1 or tubulin (loading control). The histogram shows the quantification of relative NHE-1 expression levels SD from five independent experiments. Lane 3 was defined as 1 (*). (C) Migri-NHE-1 or empty Migri vector were transduced into wild-type or pretumourigenic *CD45^−/−^Lck^F505^* thymocytes. 72 h after the first round of infection, cells were immunoblotted for NHE-1 and Bcl-x_L_. NHE-1 expression levels (NHE-1 relative intensity) were normalised for loading using tubulin values. Deamidation was calculated as in Figure 1B. The lower left FACS histogram shows the infection efficiency for nontransfected (non), empty-vector transfected (vector), or NHE-1 transfected (NHE-1) cells as percentage GFP-positive cells. The lower right histograms show the mean pH_i_ and apoptosis (sub-G1) values ± SD (*n* = 5) analysed on GFP-negative and positive cells. (D) The NHE-1 inhibitor DMA blocks DNA damage-induced alkalinisation (top left panel), Bcl-x_L_ deamidation (lower panel) and apoptosis (top right panel) in wild-type thymocytes. Thymocytes were treated with Etoposide for 24 h, or exposed to 5 Gy of irradiation and then maintained in culture for 24 h, with or without 200 μM DMA. pH_i_ was measured by FACS on live CD4^−^CD8^−^ cells, and the sub-G1 peak was analysed by FACS on CD4^−^CD8^−^ cells to assess apoptosis. The histograms represent mean values ± SD (*n* = 3).

We next investigated the molecular mechanisms leading from DNA damage to the regulation of pH_i_ and subsequent Bcl-x_L_ deamidation. Figure 4A shows that de novo protein synthesis is essential for Bcl-x_L_ deamidation following DNA damage in wild-type thymocytes. Because the NHE-1 Na/H antiport is a well-established regulator of pH_i_ [22] and has previously been implicated in the regulation of thymic apoptosis [23], we measured its expression in wild-type thymocytes after DNA damage and found that the NHE-1 level increased 2.5-fold within 5 h, whereas this increase was completely suppressed in pretumourigenic thymocytes (Figure 4B). No inhibition of increased NHE-1 expression in wild-type thymocytes was observed following addition of the Z-VAD-fmk caspase inhibitor (Figure S4A) nor following depletion of Bax and Bak from the cells (Figure S4B). We therefore carried out a further series of experiments to demonstrate that there was a direct causal linkage between the regulation of NHE-1 expression, pH_i_, Bcl-x_L_ deamidation, and apoptosis. Given that the OTK blocks DNA-damage induced NHE-1 expression in pretumourigenic thymocytes, this provides a powerful system for examining the consequences of experimentally enforcing NHE-1 expression in these cells by retroviral transduction. As Figure 4C illustrates (upper panel), an enforced 2-fold–3-fold increase in NHE-1 expression in pretumourigenic thymocytes, without DNA damage, restored Bcl-x_L_ deamidation to a level comparable to that observed in a retrovirally transduced wild-type control in five separate experiments, thereby bypassing the OTK-mediated inhibition in deamidation. Overexpression of NHE-1 increased both pH_i_ and apoptosis to comparable levels in both pretumourigenic and wild-type thymocytes (Figure 4C, lower panels). These results suggest that increased NHE-1 expression per se is sufficient to cause increased pH_i_, Bcl-x_L_ deamidation and apoptosis. To address this question further, we used the selective NHE-1 inhibitor 5-(N,N′-dimethyl)-amiloride (DMA) to block the actions of the antiport following its increased expression on thymocytes upon DNA damage. Figure 4D shows that DMA prevented the alkalinisation of wild-type thymocytes following DNA damage (top left panel), their apoptosis (top right panel), and Bcl-x_L_ deamidation (lower panel), correlating with increased survival (Figure S5A).

**Figure 5:**
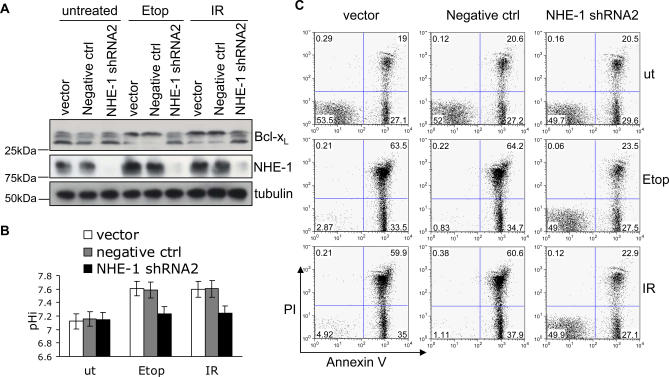
NHE-1 Knockdown Blocks DNA Damage–Induced Bcl-x_L_ Deamidation and Apoptosis (A) Empty vector, negative control, or NHE-1shRNA2 were transduced into wild-type thymocytes, then treated with Etoposide (Etop) or irradiation (IR) prior to immunoblotting for NHE-1 and Bcl-x_L_. (B) Aliquots of the cells from (A) were analysed for pH_i_. The histogram represents mean values ± SD (*n* = 3). (C) Aliquots of the cells from (A) were analysed for apoptosis by Annexin V/PI staining using flow cytometry, as illustrated in a representative experiment (total *n* = 5). The numbers shown are the percentage of cells in each quandrant. Histograms summarising the percentage of apoptotic cells (Annexin V^+^PI^−^) and dead cells (Annexin V^+^PI^+^) are shown in Figure 6B.

To extend these findings, we also used shRNA to deplete thymocytes of NHE-1 protein (Figure S6A). NHE-1 knockdown almost completely blocked the actions of DNA damage in causing Bcl-x_L_ deamidation (Figure 5A), intracellular alkalinisation (Figure 5B), or apoptosis (Figure 5C and Figure S6B). We measured apoptosis by two different methods to ensure that DNA damage–induced cell death following retroviral transduction was by apoptosis and not by necrosis. Figure 5C and Figure S6B illustrate that double staining for Annexin V and propidium iodide (PI) followed by FACS analysis revealed a major increase in Annexin V^+^ PI^−^ (apoptotic) cells following transduction with the negative control shRNA followed by either γ irradiation or treatment with etoposide, whereas there was no increase in apoptotic cells above baseline in the cells depleted of NHE-1: DNA damage–induced apoptosis was blocked 100%. Comparable results were obtained by measuring the sub-G1 peak by FACS (unpublished data) and NHE-1 depletion also correlated with increased survival (Figure S5B).

**Figure 6:**
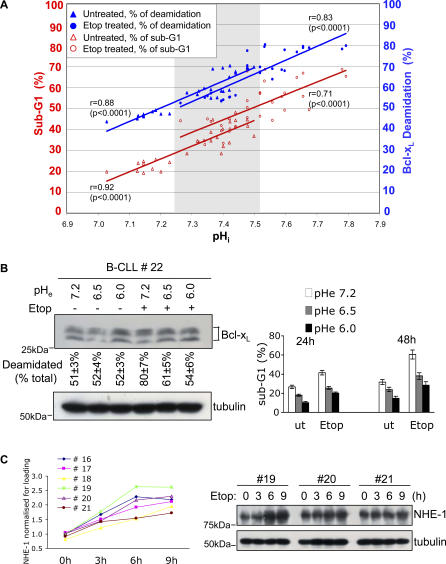
DNA Damage Induces NHE-1 Expression, and Enforced Alkalinisation Promotes Apoptosis of Human B-CLL cells (A) Enforced alkalinisation of cancer cells from patients (*n* = 10) with B-CLL causes Bcl-x_L_ deamidation and associated cell death. Treatment with etoposide (Etop) in vitro further amplifies cell death. Patients' cells (PBMC, in the range 85%–95% CD19^+^B220^+^) were incubated at pH_e_ values of 7.2, 8.0, or 8.5, and the pH_i_ values were monitored by SNARF-1 staining using flow cytometry. Apoptosis was evaluated by measurement of sub-G1 peaks using flow cytometry. The data shows pooled results from ten patients via 30 values per treatment condition: due to identical values, some symbols overlap. The correlation coefficients (*r*) of deamidation or sub-G1 versus pH_i_ are shown for each treatment. The *p* value (significance) for each correlation is shown in parentheses. The correlation coefficients of sub-G1 versus deamidation are *r* = 0.92 (*p* < 0.0001) for untreated cells and *r* = 0.87 (*p* < 0.0001) for etoposide treated cells. (B) Purified PBMC from B-CLL patients were cultured for 24 h in media at the pH shown, with/without etoposide for 48 h, and then analysed for Bcl-x_L_ deamidation by immunoblotting (left panel). The upper and lower bands were quantified and the upper deamidated Bcl-x_L_ band was expressed as a percentage of total Bcl-x_L_. The percentages shown below each lane are means ± SD (*n* = 4). The same cell aliquots cultured in RPMI/10% FCS for 24 h or 48 h were analysed for apoptosis by sub-G1 staining (right panel). (C) Assessment of NHE-1 expression in B-CLL patients' samples following exposure to etoposide for the times shown. Representative immunoblotting results are shown for three patients in the right panel and the values for six patients (normalized for tubulin loading) are graphed in the left panel.

We considered that post-translational modification of the NHE-1 antiport, in addition to regulation of its expression, might also be involved in mediating the DNA damage response. For example, a number of serine kinases have been shown to regulate NHE-1 phosphorylation and activity [24,25], so we investigated the pSer and pThr levels in NHE-1 immunoprecipitates from irradiated wild-type and pretumourigenic thymocytes, but the basal level of phosphorylation did not change after DNA damage and was comparable between the two cell types (Figure S6C). Nevertheless, we cannot formally exclude the possibility that not all pSer/pThr sites were recognised by the cocktail of monocolonal antibodies (mAbs) used. Taken together, our findings therefore suggest that the increased expression of the NHE-1 transporter is both necessary and sufficient for DNA damage–induced alkalinisation, Bcl-x_L_ deamidation, and apoptosis in wild-type thymocytes, and that the suppression of these three parameters in pretumourigenic thymocytes is caused by oncogenic inhibition of the DNA damage–triggered increase in NHE-1 expression.

### Enforced Alkalinisation Causes Increased Bcl-x_L_ Deamidation and Apoptosis in Murine and Human Cancer Cells

The experiments illustrated in Figures 1–5 were all carried out on wild-type or primary pretumourigenic *CD45^−/−^lck^F505^* thymocytes. Signalling pathways can be markedly different in fully transformed cells compared to their pretransformed counterparts. We therefore wondered whether *CD45^−/−^lck^F505^* T cell tumour cells, which develop from CD4^−^CD8^−^ thymocytes [16], might display a comparable set of properties. Figure S7 shows that this was indeed the case: murine tumour cells resistant to genotoxic insult at physiological pH_i_ values can be sensitised to die by enforced alkalinisation leading to Bcl-x_L_ deamidation. Furthermore, a modest rise in pH_i_ following incubation in a mildly alkaline buffer produces levels of Bcl-x_L_ deamidation and apoptosis in murine tumour cells comparable to those observed by adding a DNA damaging reagent to wild-type thymocytes incubated at physiological pH.

Chronic lymphocytic leukaemia (CLL) is the most common adult haematological malignancy in the Western world and, like many cancers, is characterised by the development of drug resistance. We therefore determined whether genotoxic treatment in vitro of primary human B lineage CLL (B-CLL) cells might cause increased NHE-1, alkalinisation, Bcl-x_L_ deamidation, and apoptosis, as in primary murine thymocytes (Figures 3–5), or whether this might be inhibited, as with the murine cancer cells (Figure S7). In addition, we examined the consequences for these parameters of incubating cancer cells in alkaline pH buffers. To perform these investigations, we divided each sample of patient cancer cells into nine aliquots that were either untreated, subjected to γ irradiation, or exposed to etoposide, followed by incubation at pH 7.2, pH 8.0, or pH 8.5 for 24 h. Each aliquot was then further subdivided into three samples to measure pH_i_, Bcl-x_L_ deamidation, and apoptosis. As expected, exposure of cells to mildly alkaline buffers generated pH_i_ values that displayed some variation between samples from different patients within a narrow range. The 18 values per patient obtained from 10 different patients, the mean values calculated for each pH_e_ value considered separately, and representative Bcl-x_L_ deamidation results from a single patient are illustrated in Figure 6A, Figure S8A, and Figure S8B, respectively. Interestingly, unlike the murine tumour cells expressing an OTK, the B-CLL cells behaved somewhat more like wild-type thymocytes in that DNA damage at physiological pH_e_ caused a mean increase of pH_i_ of 0.22 units, an 8% increase in Bcl-x_L_ deamidation, and an 18% increase in the number of cells undergoing apoptosis (Figure 6A and Figure S8A), compared to the higher thymocyte values of 0.45 pH_i_ units, 40% increase, and 37% increase, respectively (Figure 3). The human cancer cell values for these parameters were greatly increased at alkaline pH_e_, generating tight correlations between increasing pH_i_, Bcl-x_L_ deamidation, and apoptosis (*r* values shown in Figure 6A). Thus, a mean increased pH_i_ of 0.5 correlated with 1.7-fold and 2.4-fold increases in Bcl-x_L_ deamidation and apoptosis, respectively. It is also striking that enforced intracellular alkalinisation alone (by 0.3 pH_i_ units), in the absence of experimentally induced DNA damage, was itself sufficient to increase Bcl-x_L_ deamidation and apoptosis by 1.5-fold and 1.8-fold, respectively. This point is further illustrated by the gray shaded area shown in Figure 6A, which encompasses the overlap in sub-G1 (apoptosis) values that were obtained either by DNA damage at physiological pH or by enforced alkalinisation without DNA damage. Conversely, incubation of B-CLL cells at lower pH inhibited DNA damage–induced Bcl-x_L_ deamidation and apoptosis (Figure 6B). Therefore with respect to enforced changes in pH_i_, the B-CLL cells behaved in a comparable way to both murine thymocytes and tumour cells. A small increase in pH_i_ induced by incubation in alkaline buffer in the absence of induced DNA damage generated as much, if not more, Bcl-x_L_ deamidation and apoptosis as that triggered by genotoxic attack at physiological pH_e_.

NHE-1 expression in response to DNA damage was investigated in a further six B-CLL patients. Figure 6C shows by immunoblotting (right panel) that there was some variation between patients, but that in all cases (left panel), etoposide caused increased NHE-1 expression by 3 h, achieving optimal values by 6–9 h ranging from 1.9-fold–2.6-fold over basal levels. These increases correlate with the observed increases in Bcl-x_L_ deamidation and apoptosis in patients' cells (Figure 6A) and at the 2.6-fold level, at least, are comparable with the increases observed in wild-type thymocytes (Figure 4B). Furthermore, DNA damage–induced Bcl-x_L_ deamidation in B-CLL cells was prevented by addition of either cycloheximide (CHX) (Figure S8C) or DMA (Figure S8D), establishing a possible linkage between DNA damage, NHE-1 function, and Bcl-x_L_ deamidation in human cancer cells.

## Discussion

It has previously been suggested that Bcl-x_L_ deamidation is critical in the signalling pathway that leads from DNA damage to apoptosis [9]. This interpretation was based to a large degree on the observation that N52D/N66D Bcl-x_L_, one of the species generated by deamidation, can no longer exert anti-apoptotic activity nor sequester the pro-apoptotic protein Bim. However, a secondary mutation in the N52D/N66D Bcl-x_L_ construct was later discovered, which, when corrected, restored binding, thereby casting doubt on the initial interpretation of the physiological significance of Bcl-x_L_ deamidation [13]. We now propose that the initial finding was correct, but for the wrong reason. Our results indicate that the major Bcl-x_L_ species generated by deamidation in situ is not Asp^52^/Asp^66^ but iso-Asp^52^/iso-Asp^66^, which is consistent with the well-established biochemistry of Asn deamidation [1], and that this species is unable to sequester Bim or Puma (Figure 2 and Figure S2). The introduction of iso-Asp into the disordered loop in which these residues are located is expected to cause greater conformational change than Asp, because of the redirection of the peptide backbone through β carboxyl groups, as indicated by the known structural and functional changes that occur in proteins upon conversion of Asn to iso-Asp residues [26,27]. The structural importance of protein iso-Asp residues is likewise underlined by the expression of the putative repair enzyme L-isoaspartate O-methyltransferase which converts iso-Asp to Asp residues: its deletion has striking effects on protein functions [28–30]. Furthermore, comparison of the crystal structures of native rat Bcl-x_L_ with its deamidated version has revealed significant differences [10]; the structural implications of introducing iso-Asp residues into the disordered loop environment of Asn^52^/Asn^66^ merits further work.

**Figure 7:**
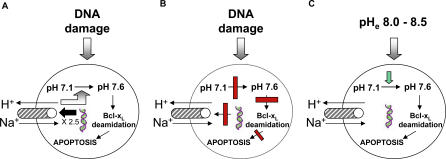
Models Illustrating the Linkage Between DNA Damage, the NHE-1 Antiport, Alkalinisation, Bcl-x_L_ Deamidation, and Apoptosis in Wild-Type and Cancer Cells (A) In wild-type thymocytes, DNA damage causes increased NHE-1 expression and a consequent rise in intracellular pH, Bcl-x_L_ deamidation, and apoptosis. (B) In pretumourigenic thymocytes expressing an OTK, the DNA damage–induced rise in NHE-1 expression is blocked, preventing alkalinisation, Bcl-x_L_ deamidation, and apoptosis. (C) Enforced alkalinisation of murine tumour cells, or human B-CLL cells, causes Bcl-x_L_ deamidation and subsequent apoptosis, even in the absence of external genotoxic attack.

We have identified critical elements in the signalling pathway leading from DNA damage to Bcl-x_L_ deamidation in thymocytes and have shown, as Figure 7A illustrates, that deamidation is induced upon DNA damage by up-regulation of the NHE-1 antiport and consequent intracellular alkalinisation (Figures 3–5). To the best of our knowledge, this represents the first description of a molecular mechanism for the regulation of protein internal Asn deamidation in cells. Our results are consistent with the failure, until now, to identify genes encoding internal protein Asn deamidases [1]. The regulation of NHE-1 antiport function is complex, involving modulation of its expression, phosphorylation, and binding of regulatory proteins [24,25,31]. Our data are consistent with a model in which DNA damage causes alkalinisation by a direct 2–3-fold increase in NHE-1 expression (Figures 4B and 6C), although we cannot exclude the possibility that undetected changes in phosphorylation might shift the pH dependence of the antiport to a more alkaline range as described for myocardial tissue [32]. Furthermore, the calcineurin B homologous protein 1 (CHP-1) has been characterised as an essential cofactor for NHE-1 in normal tissues [33], whereas its CHP-2 homologue is up-regulated in transformed cells [34], so regulation of these proteins might also be involved in activation of the antiport. Intracellular NHE-1 mediated alkalinisation has previously been implicated in the regulation of HL-60 cell apoptosis [35] and in apoptosis after trophic factor withdrawal [24]. In our present work, it is clear that the alkalinising affects of DNA damage can be mimicked simply by overexpressing NHE-1 on wild-type thymocytes in the absence of DNA damage (Figure 4C). Furthermore, either inhibition or depletion of the antiport blocks DNA damage induced alkalinisation, Bcl-x_L_ deamidation, and apoptosis (Figures 4D, 5A–5C, and Figure S8D).

The direct role played by the deamidation of Bcl-x_L_ to its iso-Asp^52^/iso-Asp^66^ version in the signalling pathway from DNA damage to apoptosis is supported by the finding that either the N52D/N66D or N52A/N66A Bcl-x_L_ mutants, which still bind BH3-only proteins (Figure 2E), protect thymocytes from dying upon enforced intracellular alkalinisation (Figure 3G). An alternative hypothesis involves the generation of new BH3-only family members as a consequence of alkalinisation, which compete for binding to Bcl-x_L_, thereby displacing Bim and Puma. However, such a hypothesis does not explain why the Bcl-x_L_ mutants that still bind BH3-only proteins retain their anti-apoptotic potency at high pH (Figure 3G).

The striking blockade in DNA damage–induced NHE-1 expression, alkalinisation, Bcl-x_L_ deamidation, and apoptosis noted in *CD45^−/−^lck^F505^* pretumourigenic thymocytes (Figures 3 and 4), together with the reversal of this blockade by enforced expression of NHE-1 (Figure 4C), provide strong support for the model illustrated in Figure 7B. The oncogenic hyperactive p56^lck-Y505F^ tyrosine kinase [15] must inhibit one or more steps on the pathway from DNA damage to increased NHE-1 expression, a mechanism that is under active investigation. We have previously demonstrated in pretumourigenic thymocytes a tight correlation between inhibition of Bcl-x_L_ deamidation, resistance to DNA damage induced apoptosis, and oncogenesis, suggesting that the consequent accumulation of DNA-damaged thymocytes is critical in the transforming process [14,15]. It therefore seems conceivable that the OTK-induced inhibition of NHE-1 is likewise important in thymic transformation, and further in vivo work will be necessary to investigate this possibility.

The resistance to genotoxic attack by *CD45^−/−^lck^F505^* murine tumour cells correlates, as in their pretumourigenic counterparts, with the inhibition of DNA damage–induced NHE-1 antiport expression, alkalinisation, Bcl-x_L_ deamidation, and apoptosis (Figure S7), which is an apparent example of ”oncogene addiction”, whereby oncogene expression continues to be important for survival [36]. By contrast, DNA damage of human B-CLL cells, which should not express OTKs, triggered increased NHE-1 expression and apoptosis, achieving levels comparable with wild-type thymocytes (Figure 6C). However, enforced alkalinisation of either the murine (Figure S7) or human (Figure 6) cancer cells triggered significant increases in Bcl-x_L_ deamidation and apoptosis, even in the absence of genotoxic attack (Figure 7C). In the case of the B-CLL cells, we cannot yet exclude the possibility that the tight correlation observed between these events does not reflect causal efficacy, and further work will be necessary to elucidate this point. In any event, the key issue for cancer cell therapy in this context is not whether inhibition of Bcl-x_L_ deamidation is involved in the initial transforming process, but whether Bcl-x_L_ is the main prosurvival protein protecting the tumour cells from the normal consequences of DNA damage. An extensive literature suggests that Bcl-x_L_ does indeed play this role in many tumour types [37]. For example, the down-regulation of Bcl-x_L_ promoted the apoptosis of KARPAS-299 cells derived from a patient with anaplastic large cell lymphoma [38], and down-regulation of Bcl-x_L_ suppresses the tumourigenic potential of the causative NPM-ALK oncogenic fusion protein in vivo [39]. Knockdown of Bcl-x_L_ also significantly reduces the viability of pancreatic cancer cells to tumour necrosis factor α (TNF-α)– and TNF-α - related apoptosis-inducing ligand (TRAIL)-mediated apoptosis by antitumour drugs [40]. Furthermore, Bcl-x_L_ deamidation is inhibited in hepatocellular carcinomas, which are highly resistant to genotoxic treatments [11]. Our findings therefore have potential relevance to cancer therapy, whereby enforced alkalinisation, perhaps by amplification of NHE-1 expression, would promote Bcl-x_L_ deamidation, thereby triggering apoptosis.

The pioneering work of Warburg [41] established that tumours display acidic extracellular pH, although more than half a century passed before it was clearly established that the intracellular pH of tumour cells is comparable with normal cells [42]. Warburg's legacy has included intermittent interest in the possibility of pH manipulation as a means to cancer therapy. Our findings not only establish that protein deamidation can be regulated by intracellular pH change in vivo, but they also suggest that strategies for pH manipulation in antineoplastic therapy should continue to receive attention, albeit for reasons different from those envisaged by Warburg.

## Materials and Methods

### Mice.

All mice were bred and housed in specific pathogen-free conditions in the animal facility at The Babraham Institute, Cambridge, United Kingdom. The p56^Lck-F505^ (PLGF-A) transgenic mice [43] and the *CD45^−/−^* and *CD45^−/−^lck^F505^* mice have been previously described [16].

### Reagents and antibodies.

Etoposide, CHX, DMA, PI, monensin, nigericin, and goat-anti-rat immunoglobulin-agarose were from Sigma (St. Louis, Missouri, United States); protein A-sepharose and protein G-sepharose were from Amersham (Uppsala, Sweden); SNARF-1 was from Molecular Probes (Eugene, Oregon, United States); Z-VAD-fmk was from Santa Cruz Biotechnology. The following antibodies were used for Western Blotting: Bim (559685) from Pharmingen (San Diego, California, United States); Bcl-x_L_ (610212) and NHE-1 (clone 54) from Transduction Lab (New Jersey, United States); Puma (ab9643) from Abcam (Cambridge, United Kingdom); Bax (06–499) and Bak (06–536) from Upstate (New York, United States); Caspase-9 (9504) from Cell Signaling (Beverly, Massachusetts, United States); phosphoserine detection kit from Calbiochem (Darmstadt, Germany); β actin and α tubulin from Sigma.

### Recombinant Bcl-x_L_ analysis.

Image clone (2823873) containing the sequence for human Bcl-x_L_ was obtained from the MRC gene service (United Kingdom). The DNA coding amino acids 1–196 (of 233) was amplified by PCR and cloned into pENTR/D-TOPO (Invitrogen, Carlsbad, California, United States). The DNA was sequenced and the insert subcloned into pDEST17 (coding for a hexa-histidine tag) and transformed into Escherichia coli expression host DE3 (Novagen, Madison, Wisconsin, United States). Recombinant Bcl-x_L_ (His-N terminal tagged) was expressed in E. coli and purified using Co^2+^ chelation beads so that rapid elution could be performed at pH 7.0 to prevent deamidation. After anion exchange purification, three peaks (A, B, and C) were collected. Aliquots (1 μl) of each peak were desalted for mass spectrometric analysis by solid-phase microextraction on C4 Zip Tips (Millipore, Billerica, Massachusetts, United States) and the proteins eluted with 0.1% formic acid/50% aqueous acetonitrile (1 μl) directly into a nanospray tip (Protana Engineering, Odense, Denmark). The nanospray tip was inserted into a nanoelectrospray ion source (Protana Engineering) attached to a quadupole time-of-flight (TOF) mass spectrometer (Qstar Pulsar i, Applied Biosystems-MDS Sciex, Foster City, California, United States) and full scan TOF spectra were acquired at an ionization potential of 900V for 5 min over the mass/charge *(m/z)* range of 500-2000 atomic mass units. The mass spectrometric data were averaged and deconvoluted using the Bayesian Protein Reconstruct function in BioAnalyst software (Applied Biosystems). For nickel precipitation, each rBcl-x_L_ species was added to C57BL/6 thymocyte lysates for 2 h at 4 °C at pH 7.2, and Ni^2+^ beads were used to precipitate the rBcl-x_L._and complexed Bim.

### Mass spectrometric analysis of Bcl-x_L_ peptides.

Samples of native and base-treated rBcl-x_L_ (0.1 μg/μl) were digested with chymotrypsin (sequencing grade, 10 ng/μl; Roche, Basel, Switzerland) in 0.1 M ammonium acetate pH 6.2 containing 0.1% octylglucoside for 16 h at 30 °C. These digestion conditions were chosen after careful optimisation to give good and consistent yields of the peptides SDVEENRTEAPEGTESEMETPSAINGNPSW (peptide 1) and HLADSPAVNGATGHSSSL (peptide 2), containing the putative deamidation sites N52 and N66, respectively, but without inducing further deamidation. Aliquots of the digestion mixtures were analysed by liquid chromatography mass spectrometry (LC-MS) on a quadrupole TOF mass spectrometer (Qstar pulsar i, Applied Biosystems-MDS Sciex), with online separation by reversed-phase nano-LC. Peptides were eluted from the column (0.075 mm × 100 mm, Vydac C18) with a gradient of 5%–35% acetonitrile (containing 10 mM ammonium acetate pH 5.3) over 30 min at a flow rate of 250 nl/min. During the development phase of the methodology, the mass spectrometer was operated in MS/MS mode to conclusively identify the peptide digestion products and to confirm the sites of deamidation as N52 and N66. Once the identities of the peptides had been established, the mass spectrometer was operated in MS mode for subsequent analyses.

For relative quantification of specific peptides, peak areas were obtained from extracted ion chromatograms of the monoisotopic mass of the corresponding pseudomolecular ions. These were: 816.60 ([M+4H]^4+^ peptide 1), 816.85 ([M+4H]^4+^ peptide 1 deamidated), 574.28 ( [M+3H]^3+^ peptide 2), and 574.61 ( [M+3H]^3+^ peptide 2 deamidated). The chromatographic conditions used for the separation of the peptides in the LC-MS analyses were optimised so as to resolve the Asn, Asp, and iso-Asp forms of peptides 1 and 2. The Asp and iso-Asp forms of the two peptides were identified by spiking an aliquot of a digestion mixture with Asp- or iso-Asp–containing synthetic peptides prior to LC-MS.

### DNA damage treatments.

Freshly isolated thymocytes were irradiated with 10 Gy using a caesium source or treated with etoposide in DMSO at a concentration of 25 μM for murine cells, or 50 μM for B-CLL cells, for the times indicated. Carrier DMSO was added to control cells.

### Immunoblotting and immunoprecipitation.

Cells were lysed in 50 mM HEPES (pH 7.2), 150 mM NaCl, 1mM EDTA, 0.2% NP-40, and complete protease inhibitors. Cell lysates were resolved by standard Laemmli's SDS-PAGE (pH 8.8) unless otherwise stated. For immunoprecipitations: rat Bim antibody (Oncogene, San Diego, California, United States) was coated to goat-anti-rat immunoglobulin-agarose; rabbit Puma antibody was coated to protein A-sepharose; mouse NHE-1 antibody was coated to protein G-sepharose; rabbit Bcl-x_L_ antibody was coated to goat-anti-rabbit immunoglobulin-agarose. Lysates were precleared with the appropriate agarose. Quantification of immunoblots was carried out using a phosphorimager (Fuji FLA3000, http://www.fujifilm.com).

### Intracellular pH measurement.

Intracellular pH was measured using a standard ratiometric method with a pH-sensitive fluorophore SNARF-1 by flow cytometry [44]. Briefly, cells in phosphate-buffered saline (PBS) were loaded with 10 μM SNARF-1 for 40 min at 37 °C, followed by washing and incubation in PBS at room temperature for 30 min prior to measurement of pH_i_. pH calibration was carried out using high potassium buffer with 10 μM nigericin. FACS data were analysed using Flowjo software to obtain the ratio based on the Fl3/Fl2 channels. It should be noted that SNARF-1 measurements provide the average pH_i_ of the intracellular environment in a cell population including, presumably, the contribution of acidified intracellular compartments. However, even if such compartments contribute slightly to the mean pH_i_ values measured, in this work, it is the change in pH_i_ that is most important. This point was also addressed by neutralising acidic compartments using monensin in some experiments.

### Measurement of apoptosis.

Cells were stained with 20 μg/ml PI (with 50 μg/ml RNase A) and analysed by flow cytometry, gating on the CD4^−^CD8^−^ subset as necessary. The sub-G1 peak was quantified as a measure of apoptosis. In addition, apoptosis was measured using the Annexin-V-Fluos Staining Kit (Roche) according to the protocol provided. To measure the percentage of dead cells, PI was used at 0.5 μg/ml.

### Generation of Bcl-x_L_ mutants.

Mouse Bcl-x_L_ cDNA was kindly provided by S. Korsmeyer (Howard Hughes Medical Institute, Harvard Medical School, Boston, Massachusetts, United States). N52A-N66A and N52D-N66D mutants were made using the QuickChange Site-Directed Mutagenesis Kit from Stratagene (La Jolla, California, United States) according to the instructions provided. The sequences of the constructs were confirmed by DNA sequencing.

### Retroviral gene knockdown and overexpression.

Murine CD4^−^CD8^−^ thymocytes were purified and cultured in the presence of interleukin-4 (IL-4) and PdBu as described [15]. The SuppressorRetro kit was purchased from Imgenex (San Diego, California, United States), and NHE-1 shRNA sequences were designed using the “siRNA tool” from the company's website. Five selected sequences were cloned into pSuppressorRetro. The sequence of NHE-1 shRNA2 is 5′-GAAACAAAGCGCTCCATCAAC-3′. Retroviral production and infection were performed according to the protocol provided. For overexpression, NHE-1 or Bcl-x_L_ (wild-type, N52A-N66A, and N52D-N66D) cDNA were amplified with AccuPrime P*fx* DNA polymerase (Invitrogen), and cloned into Xho1 and EcoR1 sites of the multiple cloning sites of the MigRI vector [45] upstream of an internal entry site followed by enhanced gree fluorescent protein (EGFP). The sequences of the inserts were verified by DNA sequencing. The plasmids were transfected into φNX cells using Lipofectamine (Invitrogen). Viral infection of CD4^−^CD8^−^ thymocytes was performed by spinoculation (1,200 g for 90 min at 30 °C). To achieve high efficiency of gene transduction, the infection was repeated every 24 h for 2–3 d. GFP-positive cells were sorted by flow cytometry using a FACsAria.

### Bax, Bak double knockdown.

The SureSilencing shRNA kit for Bax and Bak was purchased from SuperArray (Frederick, Maryland, United States). One plasmid from each kit was screened out for the best gene ablation efficiency by transient transfection. The shRNA sequence for Bax is TCAGGATCGTCCACCAAGAA, and the shRNA sequence for Bak is GGGCTTAGGACTTGGTTTGTT. To enrich the cells transfected with both plasmids which express GFP, the GFP sequence in the shRNA:Bak plasmid was replaced by DsRed using the Sma1 restriction site before GFP and the Age1 restriction site after GFP. ShRNA:Bax-GFP and shRNA:Bak-DsRed were cotransfected into primary thymocytes using the Amaxa mouse T cell nucleofector kit (Amaxa Biosystems, Koeln, Germany). Cells positive for both GFP and DsRed were sorted by flow cytometry and used for subsequent experiments.

### B-CLL patients' cell purification.

B-CLL donor peripheral blood was centrifuged through Lymphoprep (Axis-Shield PoC, Oslo, Norway), and the interphase peripheral blood mononuclear cells (PBMCs) were harvested for subsequent experiments. The purity of PBMCs was routinely checked by staining with antibodies CD3-Cy5, CD19-Fitc, and B220-PE and was analysed by flow cytometry.

### Statistics.

The Pearson coefficient of correlation (SPSS package, Chicago, Illinois, United States) was used to analyse the correlation between variables within the same group of data.

## Supporting Information

Figure S1 DNA Damage–Induced Bcl-x_L_ Deamidation Correlates with the Kinetics of Thymic Apoptosis (A) The membrane from Figure 1C was stripped and reprobed with caspase-9 antibody. Cleaveage of caspase-9 following DNA damage was inhibited in Bax/Bak knock-down thymocytes.
(B) Wild-type thymocytes were cultured in RPMI-1640/10% bovine fetal calf serum with 25 μM etoposide for the times shown, and aliquots of cells from each time point were stained with 7-AAD and analysed by flow cytometry to estimate the percentage of cells undergoing apoptosis (sub-G1 peak expressed as a % of total cells). The data illustrate a representative experiment and the mean values ± SD from five independent experiments are quantified in (B) (blue bars).
(C) Aliquots of cells from the experiments shown in (A) were analysed for Bcl-x_L_ expression by immunoblotting, and the membrane was reprobed with tubulin (loading control). The upper bands (deamidated) and lower band (native) of Bcl-x_L_ were quantified using a phosphorimager, and the percentages of upper bands in comparison to the total (upper plus lower bands) were calculated. The mean values ± SD from five independent experiments are shown in the histogram (red line).
(976 KB TIF)

Figure S2 Deamidation Disrupts the Sequestration of BH3-Only Proteins by Bcl-x_L_
(A) Puma binds to the native but not deamidated form of Bcl-x_L_. Either wild-type (1.5 × 10^7^, lanes 3 and 4) or pretumourigenic *CD45^−/−^Lck^F505^* thymocytes (1.5 × 10^7^, lanes 5 and 6) were treated as in Figure 2A, and cells were lysed and subjected to immunoprecipitation with Puma antibody, followed by blotting with either Bcl-x_L_ or Puma antibodies. Lane 1 is a wild-type thymocyte whole cell lysates (WCLs) control to facilitate comparison of native and deamidated forms of Bcl-x_L_. The asterisk indicates the light chain of the Puma antibody used for immunoprecipitation.
(B) Deamidated Bcl-x_L_ from alkali treated thymocytes no longer binds to Bim. Wild-type thymocytes were incubated in neutral (pH 7.0) or alkaline (pH 9.0) buffer at 37 °C for 24 h. Bim was immunoprecipitated from WCLs and WCL samples. Bim immunoprecipitates and Bim-depleted lysates were then separated and immunoblotted for either Bcl-x_L_ or Bim.
(944 KB TIF)

Figure S3 The Asp and iso-Asp Forms of Bcl-x_L_ Chymotryptic Peptides 1 and 2 Were Identified by Spiking an Aliquot of a Digestion Mixture with Asp- or iso-Asp–Containing Synthetic Peptides Before LC-MS Peptides SDVEENRTEAPEGTESEMETPSAINGNPSW (peptide 1) and HLADSPAVNGATGHSSSL (peptide 2) and the corresponding deamidated forms, which contain the putative deamidation sites N52 and N66, respectively, were generated by digestion of rBcl-x_L_ with chymotrypsin. The chromatographic conditions used for the separation of the peptides in the LC-MS analyses were optimised so as to resolve the Asn, Asp, and iso-Asp forms of peptides 1 and 2. The Asp and iso-Asp forms of the two peptides were identified by spiking an aliquot of a digestion mixture with Asp- or iso-Asp–containing synthetic peptides prior to LC-MS as shown. The chromatograms show LC-MS analyses at time point 72 h of the rBcl-x_L_ base treatment.
(1.1 MB TIF)

Figure S4 DNA Damage–Induced NHE-1 Up-Regulation Is Mitochondrial Apoptosis–Independent (A) Aliquots of the cells from Figure 1A incubated in the presence or absence of Z-VAD-fmk (200 μM) were analysed for the expression of NHE-1 and tubulin (as loading control) by immunoblotting.
(B) Aliquots of the cells from Figure 1C were analysed for the expression of NHE-1 by immunoblotting. Tubulin was reprobed as loading control.
(645 KB TIF)

Figure S5 Thymocytes Treated with DMA or Transduced with NHE-1 siRNA Display a Survival Advantage In Vitro Following DNA Damage (A) Purified double-negative (DN) thymocytes treated with/without DMA, etoposide, or irradiation were cultured in vitro. At 24 h, 48 h, or 72 h, an aliquot of cells was analysed by PI staining (0.5 μg/ml) using flow cytometry; PI-positive cells represent dead cells.
(B) Purified DN thymocytes transduced with NHE-1 shRNA2 or empty vector were treated with or without etoposide and irradiation and then cultured in vitro. At 24 h, 48 h, or 72 h, an aliquot of cells was analysed as in (A).
(431 KB TIF)

Figure S6 Supplementary Information for NHE-1 Knockdown and Phosphorylation Analysis. (A) Knockdown of NHE-1 by shRNA. NHE-1 shRNA (shRNA1–5), negative control, and empty vector were transduced into wild-type thymocytes. Immunoblotting for NHE-1 and tubulin showed that shRNA2 is the most potent shRNA2 inhibiting NHE-1 expression; soshRNA2 was used in subsequent experiments.
(B) The histograms summarise the percentage of apoptotic cells (Annexin V^+^PI^−^) and dead cells (Annexin V^+^PI^+^) from the experiment illustrated in Figure 5C. The data are means based on five independent experiments.
(C) The Ser phosphorylation of the NHE-1 antiport remains unchanged following DNA damage. Wild-type or *CD45^−/−^Lck^F505^* thymocytes were exposed to 5 Gy of irradiation and maintained in culture for the times shown. NHE-1 immunoprecipitates were then immunoblotted for p-Ser (16B4). The membrane was stripped and reprobed for total NHE-1. The histogram shows the relative quantification of p-Ser ±SD from three independent experiments. Lane 1 was defined as 1 (*). Note that immunoblotting with one additional p-Ser antibody and two additional p-Thr antibodies gave comparable results to those shown here.
(1.0 MB TIF)

Figure S7 Primary Tumour Cells Are Resistant to DNA Damage–Induced Bcl-x_L_ Deamidation and Apoptosis, but Enforced Alkalinisation Overcomes this Resistance. (A) DNA damage–induced Bcl-x_L_ deamidation is inhibited in *CD45^−/−^Lck^F505^* tumour cells. Wild-type, *CD45^−/−^Lck^F505^* pretumourigenic, and *CD45^−/−^Lck^F505^* tumour cells were either treated with etoposide for 24 h or exposed to 5 Gy of irradiation and then cultured for 24 h. Cells were lysed and subjected to immunoblotting for Bcl-x_L_ or tubulin (loading control).
(B) Intracellular alkalinisation and apoptosis induced by DNA damage are both inhibited in *CD45^−/−^Lck^F505^* tumour cells. pH_i_ (upper panel) and apoptosis (lower panel) were analysed as in Figure 3A and Figure 1A.
(C) DNA damage causes up-regulation of NHE-1 in wild-type but not in *CD45^−/−^Lck^F505^* tumour cells. Wild-type thymocytes or *CD45^−/−^Lck^F505^* tumour cells were either treated with etoposide (Etop) for 5 h or exposed to 5 Gy of irradiation and then maintained in culture for 5 h, followed by immunoblotting for NHE-1 or tubulin. The histogram shows the quantification of NHE-1 expression from five independent experiments SD. Lane 3 was defined as 1(*).
(D) *CD45^−/−^Lck^F505^* tumour cells were cultured in the media with the pH_e_ as shown without monensin, treated with irradiation or etoposide, and analysed for Bcl-x_L_ deamidation by immunoblotting. The percentage deamidation was calculated as in Figure 1B.
(E) Aliquots of the cells used for (D) were assessed for pH_i_ .
(F) Aliquots of the cells used for (D) were assessed for apoptosis. The histograms represent mean values ± SD (*n* = 3).
(A) shows that Bcl-x_L_ deamidation following DNA damage was suppressed in primary tumour cells to the same extent as in pretumourigenic thymocytes 24 h after inducing DNA damage, although after 48 h, the inhibition of deamidation was somewhat less (68.1% ± 5.2% inhibition in tumour cells compared to 96.2% ± 3.8% in pretumourigenic thymocytes, unpublished data). Likewise, alkalinisation (B, upper panel), apoptosis (B, lower panel) and increased NHE-1 expression (C) were all suppressed in tumour cells to nearly the same extent as in pretumourigenic thymocytes. Furthermore, in the absence of monensin, extracellular buffers at pH 8.0–8.5 forced pH_i_ values of 7.5–7.7 (E) triggering Bcl-x_L_ deamidation (D) and apoptosis (F). It is particularly striking that incubation in buffer at pH 8.0, for example, which achieves a pH_i_ value of 7.43, triggers 66.4% and 36.6% levels of Bcl-x_L_ deamidation and apoptosis, respectively, irrespective of whether, in addition, DNA damage was induced by etoposide or by γ irradiation. These results show that murine tumour cells resistant to genotoxic insult at physiological pH_i_ values can be sensitised to die by enforced alkalinisation leading to Bcl-x_L_ deamidation.
(1.3 MB TIF)

Figure S8 Inhibition of NHE-1 Synthesis by CHX or Inhibition of NHE-1 Function by DMA in B-CLL Cells Blocks DNA Damage–Induced Bcl-x_L_ Deamidation (A) Replotting of data from Figure 6A to show the absolute mean values SD (*n* = 10) for Bcl-x_L_ deamidation (right panel) and apoptosis (left panel) obtained at each of the three extracellular pH values investigated. The numbers at the top of each bar represent the mean pH_i_ values measured in the cells incubated at the pH_e_ values shown.
(B) A representative Bcl-x_L_ Western blot from the B-CLL samples analysed in Figure 6A is shown.
(C) B-CLL patients' PBMCs were treated with/without CHX, etoposide, and irradiation as in Figure 4A, 48 h later cells were subjected to immunoblotting for Bcl-x_L_. A representative blot from four independent experiments is shown. Tubulin was reprobed as loading control.
(D) B-CLL patients' PBMCs were treated with/without DMA, etoposide, and irradiation as in Figure 4D, 48 h later cells were subjected to immunoblotting for Bcl-x_L_. A representative blot from four independent experiments is shown. Tubulin was reprobed as loading control.
(1.2 MB TIF)

### Accession Numbers

The GenBank (http://www.ncbi.nlm.nih.gov/Genbank) accession numbers for proteins discussed in this paper are: Bcl-x_L_ (BC019307), Bim (NM009754), NHE-1 (BC052708), and Puma (U82987).

## Abbreviations

B-CLL: B-lineage chronic lymphoblastic leukemia
CHX: cycloheximide
DMA: 5-(N,N′-dimethyl)-amiloride
DN: double negative
Etop: etoposide
FACS: fluorescence activated cell sorter
IR: irradiation
OTK: oncogenic tyrosine kinase
PBMC: peripheral blood mononuclear cells
pH_i_: intracellular pH
pH_e_: extracellular pH
PI: propidium iodide
